# Biosynthesis of Polyhydroxybutyrate with Cellulose Nanocrystals Using *Cupriavidus necator*

**DOI:** 10.3390/polym13162604

**Published:** 2021-08-05

**Authors:** Giyoung Shin, Da-Woon Jeong, Hyeri Kim, Seul-A Park, Semin Kim, Ju Young Lee, Sung Yeon Hwang, Jeyoung Park, Dongyeop X. Oh

**Affiliations:** 1 Research Center for Bio-Based Chemistry, Korea Research Institute of Chemical Technology (KRICT), Ulsan 44429, Korea; sky77@krict.re.kr (G.S.); dawoon@krict.re.kr (D.-W.J.); hr0962@krict.re.kr (H.K.); seula@krict.re.kr (S.-A.P.); seminkim@krict.re.kr (S.K.); juylee@krict.re.kr (J.Y.L.); 2 Advanced Materials and Chemical Engineering, University of Science and Technology (UST), Daejeon 34113, Korea

**Keywords:** polyhydroxybutyrate, natural polyester, cellulose nanocrystals, nanocomposites

## Abstract

Polyhydroxybutyrate (PHB) is a natural polyester synthesized by several microorganisms. Moreover, it has excellent biodegradability and is an eco-friendly material because it converts water and carbon dioxide as final decomposition products. However, the applications of PHB are limited because of its stiffness and brittleness. Because cellulose nanocrystals (CNCs) have excellent intrinsic mechanical properties such as high specific strength and modulus, they may compensate for the insufficient physical properties of PHB by producing their nanocomposites. In this study, natural polyesters were extracted from *Cupriavidus necator* fermentation with CNCs, which were well-dispersed in nitrogen-limited liquid culture media. Fourier-transform infrared spectroscopy results revealed that the additional O–H peak originating from cellulose at 3500–3200 cm^−1^ was observed for PHB along with the C=O and –COO bands at 1720 cm^−1^. This suggests that PHB–CNC nanocomposites could be readily obtained using *C. necator* fermented in well-dispersed CNC-supplemented culture media.

## 1. Introduction

Synthetic plastics are ubiquitous in modern human life. Moreover, they have a wide range of applications owing to their excellent processability, mechanical properties, and low cost. Most petroleum-based synthetic polymers have accumulated consistently because of their high resistance to nature, resulting in serious environmental pollution. Therefore, researches to develop bio-based or oil-based biodegradable materials that can substitute conventional plastics have been actively conducted.

Polyhydroxybutyrate (PHB) is one of the representative natural polyesters of the polyhydroxyalkanoate (PHA) family and is an energy-storage product synthesized by various microorganisms in vivo [1,2,3]. PHB, a thermoplastic polymer that has the advantages of good biodegradability and biocompatibility, is evaluated as an alternative polymer with high commercial value for industrial materials, drug delivery systems, and pharmaceuticals [4,5]. However, owing to the stiff and brittle characteristics of the PHB materials, their applications are limited. For commercial applications and expanding the range of applications of PHB, several studies to improve the physical properties while maintaining the advantages of PHB by making it in a blend or composites are being conducted. PHB composites incorporated with Poly(ethylene glycol) and cellulose nanowhiskers showed a larger processing window and higher elongation at break than neat PHB [6]. Plasma-treated PHB/bacterial cellulose nanocomposites showed improved mechanical properties and antibacterial activity [7]. Blended polylactic acid (PLA) and PHB composites exhibited higher toughness than neat PHB [8].

*Cupriavidus necator* is a gram-negative β-proteobacterium that has a natural biosynthetic pathway to produce PHB [9,10]. Under nutrient limitation, *C. necator* can produce PHB in granules by redirecting carbon flux exceeding 70% of its dry cell weight for energy and carbon storage [11,12,13]. *C. necator* is a promising microorganism that can be used to produce PHB from various carbon sources, including waste or non-edible substances such as waste cooking oil or agricultural wastes [14,15]. Furthermore, *C. necator* is recognized as a useful strain for developing alternative materials for petroleum-based plastics and solving the CO_2_ problem because it has the ability to produce PHB through CO_2_ fixation [16,17].

Cellulose nanocrystals (CNCs) are rod-shaped colloidal particles with a diameter and length of 3–20 nm and 50–3000 nm, respectively, and are generally prepared from cellulose by hydrolysis with sulfuric acid [18,19]. CNCs have attracted significant attention as natural fillers because of their high aspect ratio, large specific surface area, high mechanical strength, and high elastic modulus. CNCs are used as composites of other polymers to enhance the mechanical strength or increase the interfacial adhesion between materials [20,21,22]. In addition, CNCs have several advantages such as low cost owing to the high abundance of cellulose on earth, excellent biocompatibility, and eco-friendliness. Various approaches for the modification of PHB with CNCs have been developed to enhance the thermal stability, strength, and stiffness through blending or preparation of composites [23,24,25].

In this study, PHB–CNC nanocomposites were obtained through one-pot biosynthesis and nanocomposite preparation. By dispersing the CNCs well in the culture medium, the conditions were optimized to fabricate the PHB–CNC nanocomposites during the extraction of PHB biosynthesized from *C. necator*. The effects of the CNCs on bacterial cell growth and chemical composition of the extracted polymer were investigated.

## 2. Materials and Methods

### 2.1. Materials

CNCs were purchased from the Process Development Center (PDC) at the University of Maine (Orono, ME, USA). All reagents for cell culture were purchased from BD Biosciences (Franklin Lakes, NJ, USA). All chemical reagents were purchased from Sigma-Aldrich (St. Louis, MO, USA).

### 2.2. Microorganism and Culture Media

In this study, *C. necator* (KCTC 22469) obtained from the Korean Collection for Type Cultures (Jeongeup, Korea) was used for the biosynthesis of PHB. To obtain the seed culture, *C. necator* was grown in an LB broth at 30 °C for 24 h under agitation at 250 rpm. For PHB production, the seed culture (0.5 mL) was transferred to a 250 mL flask containing 50 mL of culture media, which contained 1 g/L peptone, 1 g/L beef extract, 20 g/L glucose, and 14.5 g/L NaCl. The CNCs powder was added to the culture media at a concentration of 0.01–0.1 g/L, and then the CNCs were dispersed by ultrasonic treatment using a vibra-cell ultrasonic processor (Sonics and Materials Inc., Newtown, CT, USA), followed by transferring the seed culture.

### 2.3. Extraction of PHB

PHB was recovered from *C. necator* using sodium hypochlorite [26]. For PHB extraction, 50 mL of the culture broth was centrifuged at 4000 rpm for 10 min. The cell pellet was washed twice with 10 mM phosphate-buffered saline (pH 7.4). Following centrifugation, the cell pellet was resuspended in 5 mL of sodium hypochlorite solution (13% *v*/*v*) and incubated at 30 °C for 1 h at 250 rpm. After centrifugation and removal of the supernatant, the polymer was washed twice with deionized water and once with ethanol. The extracted PHB was dried in a vacuum oven.

### 2.4. Measurement of Cell Growth

The effect of CNCs on the growth of *C. necator* was observed using a ultraviolet-visible (UV-Vis) spectrophotometer (UV-2600, Shimadzu, Tokyo, Japan). Optical density was measured at 600 nm.

### 2.5. Biological Transmission Electron Microscopy

Intracellular PHB granules were observed by biological transmission electron microscopy (bio-TEM) using an FEI Tecnai G2 F20 TWIN TMP microscope (FEI, Hillsboro, OR, USA). After fermentation, the cells were fixed in a 4% glutaraldehyde solution at 4 °C for 4 h at 4 °C and then post-fixed in 1% osmium tetroxide.

### 2.6. Fourier-Transform Infrared Spectroscopy

To characterize PHB recovered from *C. necator* fermentation, Fourier-transform infrared (FTIR) spectroscopy was performed using Nicolet iS50 (Thermo Fisher Scientific, Waltham, MA, USA). The polymers were scanned 128 times at a resolution of 4 cm^−1^ in the wavelength range of 800–4000 cm^−1^.

### 2.7. Mechanical Properties Measurement

The PHB films were prepared by solvent casting method. PHB or PHB-CNC nanocomposites extracted from *C. necator* were dissolved in chloroform and poured into the aluminum dishes at ambient temperature. The solution was dried for 5 days to evaporate chloroform, and then vacuum dried overnight to remove residual solvent. The prepared PHB films were cut into rectangular shapes (30 mm × 5 mm × 0.15 mm) for the tensile test. A universal testing machine (Model 5943, Instron, UK) was used to determine the mechanical properties of the films. Tensile properties were examined at 10 mm min^−1^ with a 50 N load cell.

### 2.8. Water Contact Angle Measurement

The water contact angle of PHB films was measured by dropping 1 μL of deionized water to the surface of the film using a contact angle analyzer DSA25 Basic (KRUSS, Hamburg, Germany).

### 2.9. Nuclear Magnetic Resonance Spectroscopy

^1^H and ^13^C nuclear magnetic resonance (NMR) spectroscopy were conducted using an AVANCE NEO 600 (Bruker, Billerica, MA, USA) at 600 MHz and 150 MHz, respectively.

## 3. Results and Discussion

### 3.1. Effect of CNCs on the Growth of C. necator

**Figure 1:**
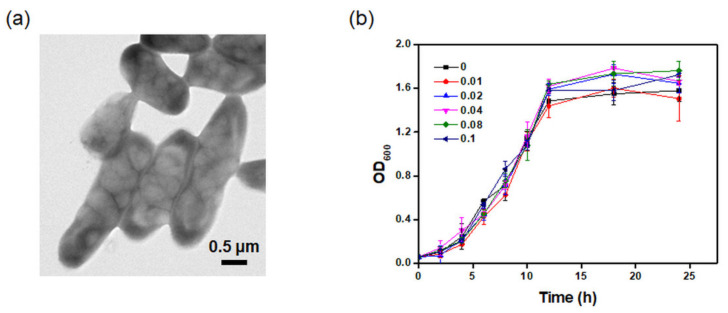
(**a**) TEM image of *C. necator* with PHB granules and (**b**) growth of *C. necator* after 24 h of incubation with different concentrations of CNCs (0–0.1 g/L).

PHB biosynthesis by *C. necator* was conducted under nitrogen-limited conditions using media containing 20 g/L of glucose, 1 g/L of peptone, and 1 g/L of beef extract. After 24 h of fermentation, the cells were harvested, and intracellular PHB was observed using TEM. The TEM image in Figure 1a shows the formation of intracellular PHB granules under the given fermentation conditions.

The development of a system to fabricate PHB–CNC nanocomposites using *C. necator* and the effect of CNCs on bacterial growth was evaluated for different concentrations of CNCs. To determine the effect of CNCs on the growth of *C. necator*, bacterial cultivation was conducted by adding CNCs in the culture media at concentrations ranging from 0 to 0.1 g/L. As shown in Figure 1b, the bacterial growth was not significantly different after 24 h of cultivation with CNCs compared to the control. This result indicates that the CNCs did not inhibit the growth of *C. necator* at a concentration of 0.1 g/L.

### 3.2. Characterization of the PHB–CNC Nanocomposite

**Figure 2:**
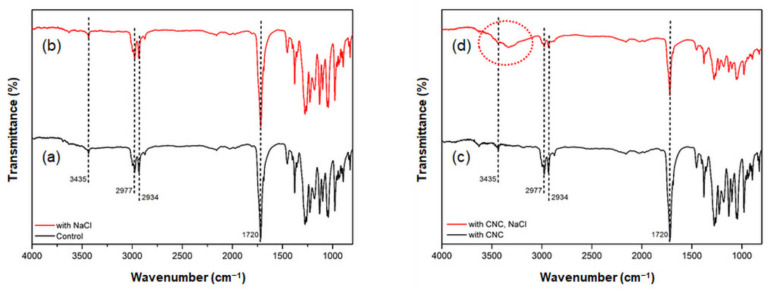
FTIR spectra of a PHB film obtained from *C. necator* incubated (**a**) without additives, (**b**) with 14.5 g/L NaCl, (**c**) with 0.1 g/L CNC, and (**d**) with 0.1 g/L CNC and 14.5 g/L NaCl.

The effects of adding CNCs and NaCl to the culture media during PHB synthesis were characterized using FTIR spectroscopy. Figure 2 shows the FTIR spectra of PHB synthesized using *C. necator* under various conditions supplemented with CNCs and NaCl. The spectra of PHB with 0.1 g/L CNC and PHB with 14.5 g/L NaCl closely matched the spectrum of the control PHB. The peak at 2934–2977 cm^−1^ corresponds to –CH, and the band at 1720 cm^−1^ indicates the presence of C=O and –COO bonds [8]. The additional broad band at 3500–3200 cm^−1^ was observed from PHB synthesized in the culture media containing 0.1 g/L CNCs and 14.5 g/L NaCl. This transmittance peak indicates the presence of O–H stretching vibration in cellulose [27]. When NaCl was added, the surface charge of both bacteria and CNCs became less negative [28]. It is speculated that the CNCs could be well-dispersed and the bacterial cells could be surrounded by the CNCs owing to weaker electrical repulsion between bacteria and CNCs.

**Figure 3:**
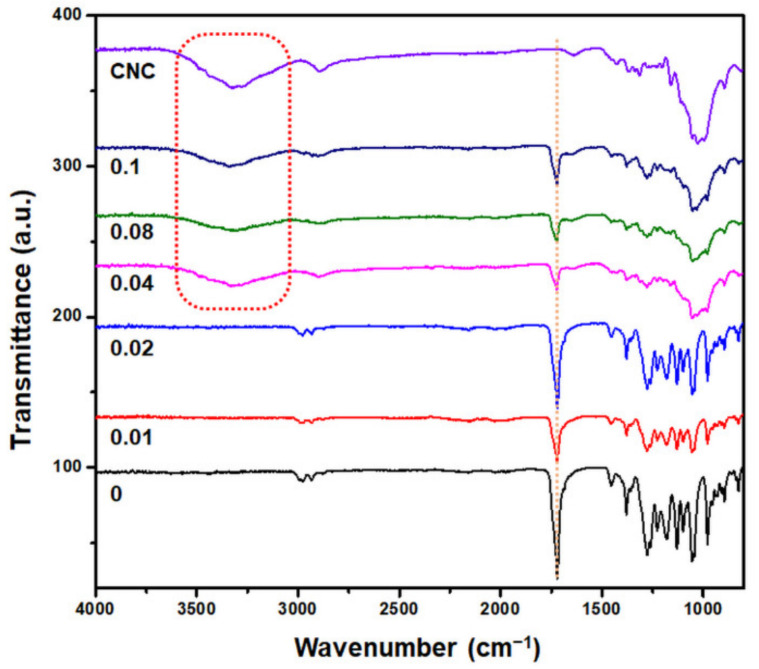
FTIR spectra of PHB synthesized by *C. necator* with different concentrations of CNCs.

To determine the effective concentration of CNCs for the preparation of PHB–CNC nanocomposite, PHB was produced supplemented with 0–0.1 g/L CNCs and 14.5 g/L NaCl. After cultivation, extracted PHB was characterized through FTIR spectroscopy. As shown in Figure 3, the PHB–CNC nanocomposites were obtained in the CNC suspension at a concentration of ≥0.04 g/L.

**Figure 4:**
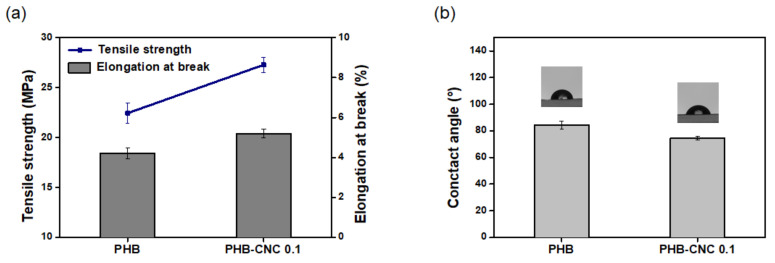
(**a**) Tensile strength and elongation at break, (**b**) water contact angle of PHB and PHB-CNC nanocomposite (0.1 g/L CNC).

Figure 4a displays the mechanical properties of PHB and PHB-CNC nanocomposite. The tensile strength and elongation at break of PHB were measured to be 22 MPa and 4%. The PHB-CNC nanocomposite fermented with 0.1 g/L CNC show the higher tensile strength (27 MPa) and elongation at break (5%) than neat PHB. Water contact angle measurements for PHB and the nanocomposite were conducted to investigate hydrophilicity on the surface. As shown in Figure 4b, the contact angle value was decreased from 84.5 ± 2.9° (neat PHB) to 74.3 ± 1.4° (PHB-CNC nanocomposite). It suggests that the incorporation of CNC increases the hydrophilicity of PHB due to the abundant hydroxyl groups of CNC. Moreover, ^1^H NMR and ^13^C NMR were used to determine the chemical structure of the polymer matrix. As shown in ^1^H NMR and ^13^C NMR spectra (Figures S1 and S2 in the Supplementary Materials), it can be seen there was no difference in the structure of polymer matrix between neat PHB and PHB-CNC nanocomposite. In other words, CNC supplement does not affect the chemical structure of PHB in the biosynthesis process.

### 3.3. Effects of CNCs Supplemented before and after Cultivation for the Nanocomposite Formation

**Figure 5:**
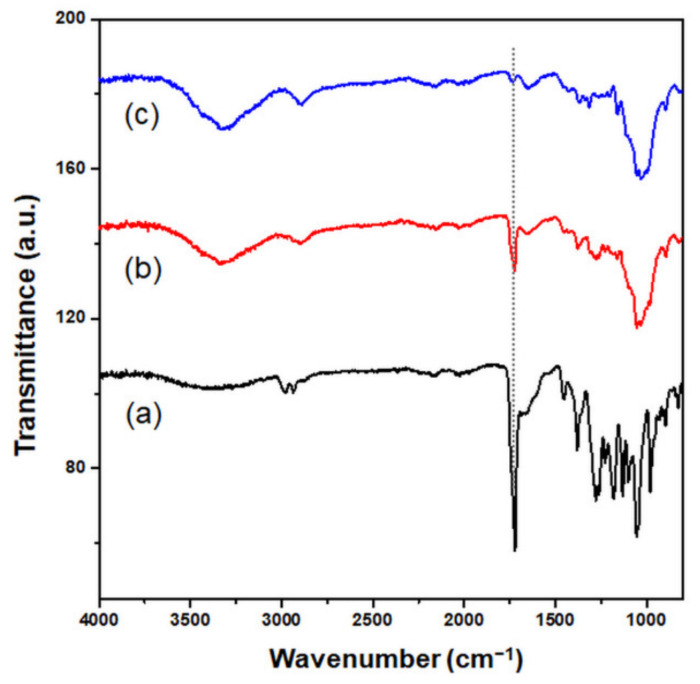
FTIR spectra of PHB produced from *C. necator* fermentation (**a**) without CNCs, (**b**) with 0.04 g/L CNCs, and (**c**) with 0.04 g/L CNCs added after cultivation.

Figure 5 shows the FTIR spectra of extracted PHBs under various conditions, that is, in the absence of CNCs, presence of 0.04 g/L CNCs, and 0.04 g/L CNCs added after fermentation. When the CNCs were added after cultivation, the FTIR spectrum of the extracted polymer showed a faint peak around 1720 cm^−1^, representing carbonyl and ester bonds. This spectrum corresponds well with the results of CNCs. This FTIR result indicates that the polymer did not form CNC nanocomposites and that the polymers, which were extracted biasedly by the CNCs or PHB, were coated with CNCs during extraction.

Comparing the FTIR peaks of the CNC-supplemented polymers before and after fermentation, it can be concluded that the PHB–CNC nanocomposites can be obtained by adding CNCs in the initial state of cultivation. It was assumed that PHB was extracted with CNCs because of the colloidal stability of the CNCs in the suspension during bacterial growth and fermentation. During cultivation, the CNCs were well-dispersed around the cell. Consequently, the PHB–CNC nanocomposites can be obtained.

## 4. Conclusions

In this study, PHB–CNC nanocomposites were obtained using CNC-dispersed culture media and PHB-producing *C. necator*. The culture conditions were optimized by adding NaCl to reduce the electrical repulsion between the CNCs and bacterial cells, thereby resulting in a well-dispersed colloid suspension. By investigating the characteristics of the extracted polymer according to the concentration of CNCs, it was confirmed that the PHB–CNC nanocomposites could be obtained when 0.04 g/L or more CNCs were added. PHB-CNC nanocomposite obtained from *C. necator* through this fermentation process showed higher mechanical properties and hydrophilicity than neat PHB. In addition, when CNCs were added after PHB fermentation, the nanocomposites could not be obtained, which is expected because CNCs cause bacterial flocculation and thus the degree of dispersion between the CNCs and bacterial cells decreases. These results suggest that synthesis of PHB-CNC nanocomposite can be achieved by applying culture media supplemented various concentrations of CNC, expanding their applications as alternative biodegradable polyester materials.

## Data Availability

The data presented in this study are available on reasonable request from the corresponding author.

## Supplementary Materials

The following are available online at https://www.mdpi.com/article/10.3390/polym13162604/s1, Figure S1: ^1^H NMR spectra of PHB (a) without CNC and (b) with 0.1 g/L CNC., Figure S2: ^13^C NMR spectra of PHB (a) without CNC and (b) with 0.1 g/L CNC.
