# Supplementary material for: Biosynthesis of Polyhydroxybutyrate with Cellulose Nanocrystals Using Cupriavidus necator
Source: Polymers (Basel). 2021 Aug 5;13(16):2604. doi: 10.3390/polym13162604 (PMC8398664; doi:10.3390/polym13162604)
Supplement: Supplementary file 1 [file polymers-13-02604-s001.zip › polymers-1289341-SI.pdf]

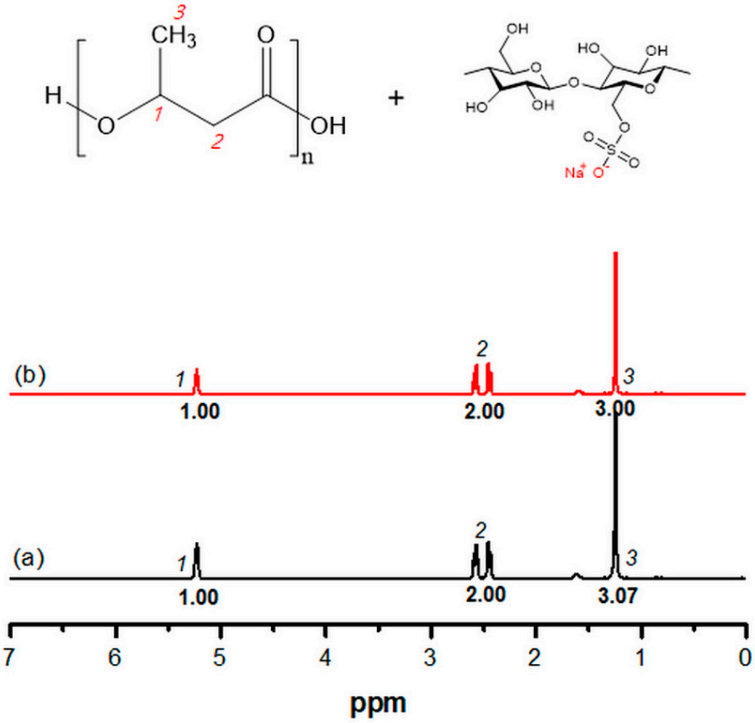

Figure S1. ^{1}H NMR spectra of PHB (a) without CNC and (b) with 0.1 g/L CNC.

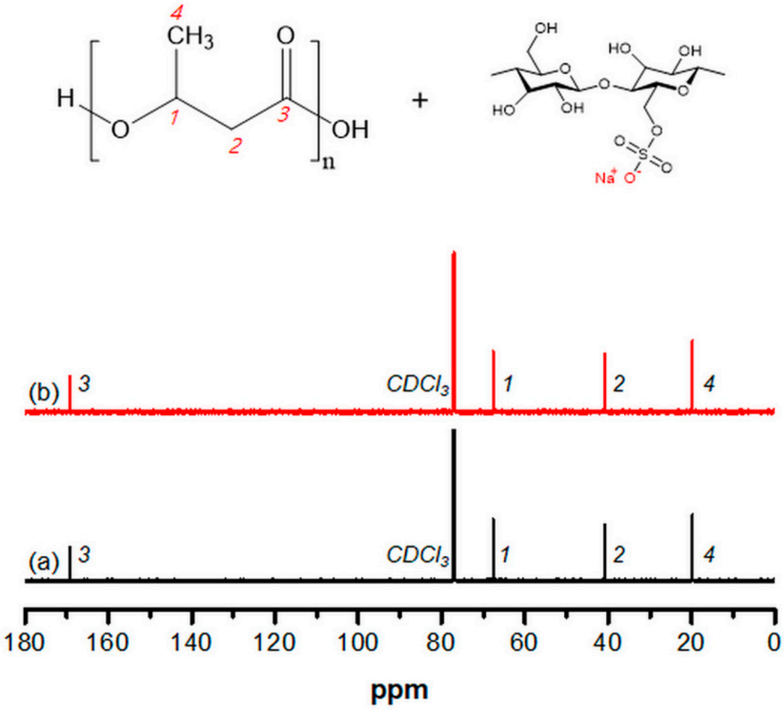

Figure S2. ^{13}C NMR spectra of PHB (a) without CNC and (b) with 0.1 g/L CNC.
